# DNA Multi-Marker Genotyping and CIAS Morphometric Phenotyping of *Fasciola gigantica*-Sized Flukes from Ecuador, with an Analysis of the *Radix* Absence in the New World and the Evolutionary Lymnaeid Snail Vector Filter

**DOI:** 10.3390/ani11092495

**Published:** 2021-08-25

**Authors:** Maria Dolores Bargues, Maria Adela Valero, Gabriel A. Trueba, Marco Fornasini, Angel F. Villavicencio, Rocío Guamán, Alejandra De Elías-Escribano, Ignacio Pérez-Crespo, Patricio Artigas, Santiago Mas-Coma

**Affiliations:** 1 Departamento de Parasitología, Facultad de Farmacia, Universidad de Valencia, Av. Vicent Andres Estelles s/n, 46100 Valencia, Spain; Madela.Valero@uv.es (M.A.V.); alejandra.elias@uv.es (A.D.E.-E.); Unho_ipc@hotmail.com (I.P.-C.); Patricio.Artigas@uv.es (P.A.); 2 Instituto de Microbiología, Edificio Eugenio Espejo, EE-125, Colegio de Ciencias Biológicas y Ambientales, Campus Cumbayá, Universidad San Francisco de Quito, Av. Diego de Robles y Vía Interoceánica, Quito 170901, Ecuador; gtrueba@usfq.edu.ec; 3 Escuela de Medicina, Universidad Internacional del Ecuador, Av. Jorge Fernández s/n y Av. Simón Bolivar, Quito 170411, Ecuador; marcovfornasini@gmail.com; 4 Departamento de Ciencias de la Vida y Agricultura, Universidad de las Fuerzas Armadas-ESPE, Sede Santo Domingo de los Tsáchilas, Vía Santo Domingo-Quevedo km 24, Santo Domingo de los Tsáchilas, P.O. Box 171-5-231B, Luz de América 230118, Ecuador; afvillavicencio1@espe.edu.ec (A.F.V.); rnguaman@espe.edu.ec (R.G.)

**Keywords:** *Fasciola hepatica*, *F. gigantica*, phenotypic characterization, morphometry by CIAS, genotypic DNA characterization, ITS-2, ITS-1, *cox*1 and *nad*1 sequencing, sheep, cattle, Ecuador

## Abstract

**Simple Summary:**

Fasciolid flukes collected from sheep and cattle in Ecuador showed a high diversity in DNA sequences whose analyses indicated introductions from South America, European and North American countries. These results agree with the numerous livestock importations performed by Ecuador. Abnormally big-sized liver flukes were found in Ecuadorian sheep. The morphometric phenotypic CIAS study showed that its size maximum and mean very pronouncedly and significantly surpassed those of the *Fasciola hepatica* populations from South America and Spain and proved to be intermediate between standard *F. hepatica* and *F. gigantica* populations. Such a feature is only known in intermediate fasciolid forms in Old World areas where the two species and their specific lymnaeid snail vectors overlap. This argues about a past hybridization after *F. gigantica* importation from Pakistan and/or introduction of intermediate hybrids previously generated in USA. The lack of heterozygotic rDNA ITS positions differentiating the two species, and of introgressed fragments and heteroplasmic positions in mtDNA genes, indicate a post-hybridization period sufficiently long as for rDNA concerted evolution to complete homogenization and mtDNA to return to homoplasmy. The vector specificity filter due to *Radix* absence should act as a driving force in accelerating such lineage evolution. Public health implications are finally emphasized.

**Abstract:**

Fascioliasis is a disease caused by *Fasciola hepatica* worldwide transmitted by lymnaeid snails mainly of the *Galba*/*Fossaria* group and *F. gigantica* restricted to parts of Africa and Asia and transmitted by *Radix* lymnaeids. Concern has recently risen regarding the high pathogenicity and human infection capacity of *F. gigantica*. Abnormally big-sized fasciolids were found infecting sheep in Ecuador, the only South American country where *F. gigantica* has been reported. Their phenotypic comparison with *F. hepatica* infecting sheep from Peru, Bolivia and Spain, and *F. gigantica* from Egypt and Vietnam demonstrated the Ecuadorian fasciolids to have size-linked parameters of *F. gigantica*. Genotyping of these big-sized fasciolids by rDNA ITS-2 and ITS-1 and mtDNA *cox*1 and *nad*1 and their comparison with other countries proved the big-sized fasciolids to belong to *F. hepatica*. Neither heterozygotic ITS position differentiated the two species, and no introgressed fragments and heteroplasmic positions in mtDNA were found. The haplotype diversity indicates introductions mainly from other South American countries, Europe and North America. Big-sized fasciolids from Ecuador and USA are considered to be consequences of *F.* *gigantica* introductions by past livestock importations. The vector specificity filter due to *Radix* absence should act as driving force in the evolution in such lineages.

## 1. Introduction

Fascioliasis is a disease included within the group of foodborne trematodiases, among the Neglected Tropical Diseases (NTDs) given priority by the World Health Organization [1,2]. The two liver flukes *Fasciola hepatica* and *F. gigantica* are highly pathogenic [3,4,5] and may underlie the underdevelopment of rural communities of low-income countries. In these depauperated rural areas, they affect mainly children [6], sometimes even very early in life [7]. In these rural areas, infected subjects usually suffer from the chronic phase of the disease, which may last for many years due to lack of diagnosis and give rise to pathological effects such as anemia, litiasis, bacterobilia, and other clinical pictures [8,9,10], which in the long term may result in sequelae [4,11]. Moreover, the immunosuppression induced by these liver flukes in both the acute and chronic phases appear linked to very frequent coinfections by other pathogenic parasites within a scenario of great morbidity [12,13,14,15].

The fasciolid flukes causing this zoonotic parasitic disease infect mammals, mainly herbivorous ruminants, but also humans that become infected by a wide spectrum of sources by oral ingestion of the infective metacercarial stage together with different foods and drinks, mainly freshwater vegetables and natural water [16]. *Fasciola hepatica* is above all transmitted by small amphibious lymnaeid species belonging to the *Galba*/*Fossaria* group in mild/cold areas and highlands of warm regions of Europe, Asia, Africa, the Americas and Oceania. *Fasciola gigantica* is transmitted by aquatic, less amphibious usually bigger lymnaeid species of the *Radix* group in warmer lowland of parts of Africa and Asia [17]. These differences in geographical distribution appear mainly related to the minimum temperature threshold for their larval stage development: 10 °C for *F. hepatica* [18,19]; 16 °C for *F. gigantica* [20,21], although cercarial shedding of the latter at 13–18 °C has been reported [22].

This disease is markedly influenced by both climate change and anthropogenic modifications of the environment [21,23], including man-made movements and import/export of livestock [24,25]. The actual spread of *F. gigantica* related to the two aforementioned phenomena, mainly global warming, is worrying, both the parasite in northern Africa [26] and the northward spread of one of its main *Radix* species vectors in Asia [27]. Indeed, *F. gigantica* has been proven to show a slower development and higher pathogenicity than *F. hepatica* [28]. Although *F. gigantica* was considered to be involved in human infection only sporadically or rarely [29,30], the discovery of human endemic zones presenting *F. gigantica* in Africa and Asia suggests the convenience of assessing the capacity of this fasciolid species to also infect humans and originate problematic public health situations. Indeed, such situations have already been described in countries as Egypt [14,31], and Ethiopia [32], in Africa, and in Iran [33], Pakistan [21], Vietnam [34], and China [35], in Asia.

The palaeobiogeographical origin of *F. gigantica* is considered to have taken place in the warm lowlands linked to ancestors of *Radix natalensis* of eastern Africa during the early Miocene, whereas *F. hepatica* should originate more recently in the highlands linked to ancestors of *Galba truncatula* of the Near East [17]. Both fasciolid species should later spread throughout Africa, Europe and Asia together with livestock along the postdomestication period, and only *F. hepatica* was later introduced into the New World and Oceania during the last 500 years as a consequence of the European colonizations [17].

In the Americas, however, the presence of *F. gigantica* or *F. gigantica*-like worms have been reported in three countries. The report in the USA [36] has been analyzed later [17]. Regarding the reports in Mexico [37,38], more recent molecular studies by ISSR-PCR found small genetic distances between liver flukes from the northwest of Spain and Mexico [39] and genetic haplotyping with the ribosomal DNA intergenic region indicated an introduction into Mexico with livestock transported from Spain during the early colonization period [40], and, the most important, the size range reported for the Mexican *F. gigantica* specimens proved to phenotypically enter in the *F. hepatica* range [40]. In South America, *F. gigantica* has been reported only from Ecuador [41], as deduced from the concrete sentence saying that “In Vietnam and Ecuador where both *F. hepatica* and *F. gigantica* are endemic......”, although no additional detail on the presence of *F. gigantica* in Ecuador is included in this article, nor in any other subsequent publication by these or other authors.

The aim of the present study is to clarify whether the aforementioned report of *F. gigantica* in Ecuador is correct or a misunderstanding, by taking advantage of the finding of liver flukes morphologically resembling *F. gigantica* in sheep from an area westward from Quito city. For this purpose, a complete phenotypic assessment and a molecular study have been performed. This study is complemented by a similar DNA multimarker analysis of fasciolid flukes collected in cattle from southern Ecuador, where uncontrolled transborder exchange of livestock occurs [42], to assess a potential introduction route from the South. In this southernmost zone of Ecuador, *Lymnaea neotropica* has recently been found [42], a very efficient vector involved in the earlier spread of fascioliasis throughout South America [43] and linked to human fascioliasis endemic areas in both the neighboring northern Peru [44] and also Argentina [45].

## 2. Materials and Methods

### 2.1. Parasites

**Figure 1:**
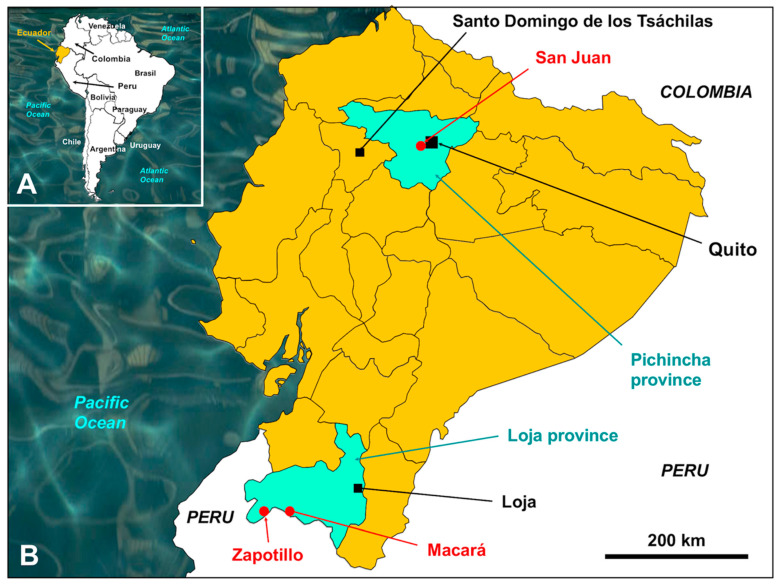
Maps showing the localities surveyed: (**A**) map of South America showing the location of Ecuador neighboring northern Peru and southern Colombia; (**B**) map showing the Pichincha province including the San Juan endemic area providing liver fluke infected sheep and cattle and Santo Domingo de los Tsachilas along the route from Quito to the Pacific coast, and the southern zone of Loja province showing the localities where cattle was surveyed in Zapotillo and Macará close to the country border with Peru.

In Ecuador, a total of 42 liver fluke specimens infecting sheep and 21 specimens infecting cattle from the zone of San Juan de Chillogallo, via Chiriboga, at an altitude of 2800–3000 m a.s.l., westward from Quito city, were obtained during slaughtering in the Camal Metropolitano de Quito. Additionally, other liver flukes infecting cattle were also obtained from the southern zone of Loja besides the border with Peru, namely 14 specimens from Macará and other 17 specimens from Zapotillo, at the lower altitudes of 480 and 153 m a.s.l., respectively [42] (Figure 1).

### 2.2. Phenotyping Analyses

#### 2.2.1. Natural and Experimental Liver Fluke Materials Used for Phenotyping Comparison

Large-sized liver fluke specimens resembling *F. gigantica* were only found in the aforementioned sheep. Previous studies on liver fluke phenotype have revealed that the definitive host species decisively influences the size of fasciolid adults and eggs [46,47,48]. Therefore, all fasciolid specimens included in the needed comparative morphometric study were gravid adult flukes (i.e., containing from few to many eggs inside the uterus) from sheep.

For this phenotyping study, liver fluke adults obtained from naturally infected sheep were studied by means of appropriate morphometric analyses by Computer Image Analysis System (CIAS) according to previously established standards [49]. Post-mortem examinations were carried out on all animals as soon as possible. The liver parenchyma and bile ducts were examined and flukes inside were collected.

A phenomenon of crowding effect on liver flukes has been emphasized in sheep [50]. Worm size is dependent on the infection level, and that worm size decreases when the burden increases [51]. Therefore, the flukes studied were only from livers with low intensity infection (<70 adults per host), to avoid a possible crowding effect influence.

The studied flukes comprised the largest worm variability possible, i.e., including different stages of body size, maturity, and gravid uteri (i.e., from smallest specimens presenting only very few eggs in the uterus, up to the largest specimens presenting plenty of eggs in the uterus; nongravid specimens presenting no eggs were not included). Fluke specimens were fixed in Bouin’s solution between a slide and coverglass, considering that coverglass pressure could not produce distortion. The worms were colored with Grenacher´s borax, and subsequently differentiated, dehydrated and mounted in Canada balsam. The following materials from natural infections were used for comparisons with the aforementioned fluke materials from Ecuadorian sheep (code: nEc):

- 47 adults of *F. hepatica* from 5 sheep (range: 3–21/sheep) from Rodicio, Cajamarca Valley, at an altitude of 2726 m a.s.l., northern Peru (nCaj);
- 130 adults of *F. hepatica* from 8 sheep (range: 2–32/sheep) from Huayucachi, Pachacayo and Huancayo, in the Mantaro Valley, at an altitude of 3231–3989 m a.s.l., Peru (nMan);
- 201 adults of *F. hepatica* collected in 12 sheep (range: 6–30 worms per sheep) from Batallas, Northern Bolivian Altiplano, at an altitude of 3858 m a.s.l., Bolivia (nAlt);
- 37 adults of *F. hepatica* from 5 sheep (range: 2–10/sheep) from Massamagrell, Valencia, Comunidad Valenciana, at an altitude of 20 m a.s.l., Spain (nSp);
- 31 adults of *F. gigantica* from 2 sheep (range: 6–32/sheep) from Giza, Giza Governorate, at an altitude of 40 m a.s.l., Egypt (nEg).

Additionally, for the same comparative phenotyping analysis, liver fluke adults of both species *F. hepatica* and *F. gigantica* experimentally obtained in sheep were also used. *Fasciola hepatica* and the lymnaeid *Galba truncatula* originated from Spain, whereas *F. gigantica* and its snail host *Radix natalensis* originated from Egypt [28]. The experimental group consisted of eight four-to-five-week-old sheep of the Guirra autochthonous breed (only sheep breed proved to be similarly susceptible to the two fasciolid species) divided into two groups of four animals each. The groups were infected per os with 200 *F. hepatica* metacercariae or 200 *F. gigantica* metacercariae, respectively. Food and water were provided ad libitum. Necropsy was carried out 40 weeks post infection. The following experimentally obtained materials were used for the same aforementioned comparative phenotyping study:

- 127 adults of *F. hepatica* from 4 experimentally infected sheep (range: 33–66/sheep; 24-week-old) from Spain (expSp);
- 100 adults of *F. gigantica* from 4 experimentally infected sheep (range: 17–69/sheep; 24-week-old) from Egypt (expEg);
- 70 adults of *F. gigantica* from 7 experimentally infected sheep (range: 2–69/sheep; 52-week-old) from Vietnam (expVi).

#### 2.2.2. Measurement Techniques

**Figure 2:**
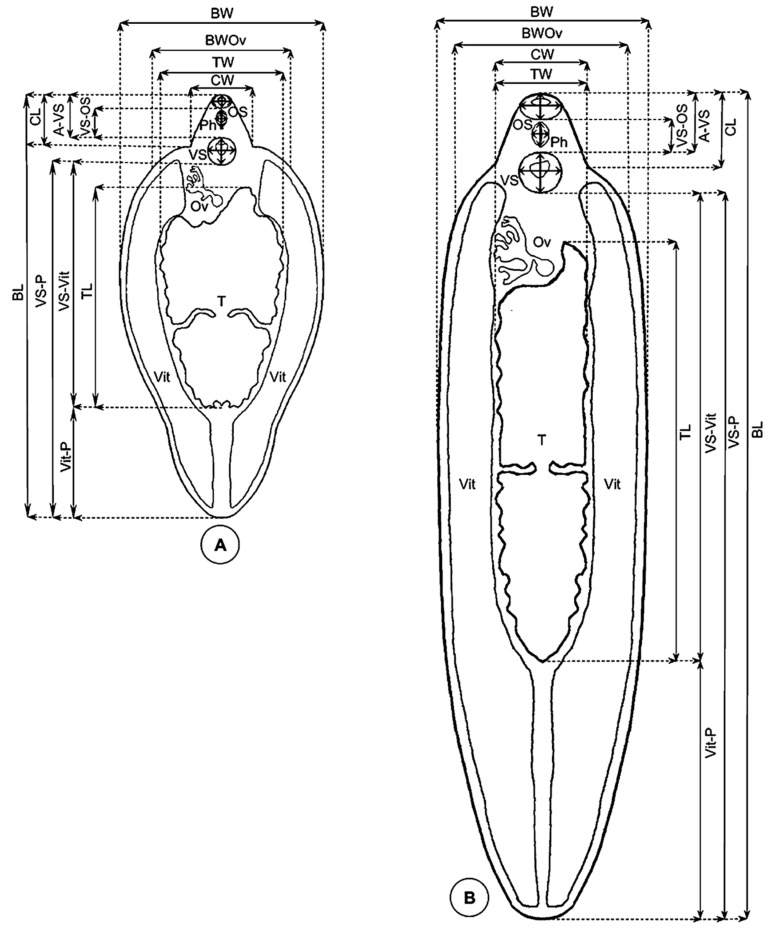
Standardized measurements in gravid fasciolid adults: (**A**) *Fasciola hepatica*; (**B**) *Fasciola gigantica.* For abbreviations, see text Section 2.2.2 (schematic drawing by M.A. Valero).

A methodology whose accuracy has been previously verified for Fasciolidae (Figure 2) [49,52,53] was applied for the obtaining of all standardized measurements of adults needed:

- Lineal biometric characters: body length (BL), maximum body width (BW), body width at ovary level (BWOv), body perimeter (BP), body roundness (BR), cone length (CL), cone width (CW), maximum diameter of oral sucker (OS max), minimum diameter of oral sucker (OS min), maximum diameter of ventral sucker (VS max), minimum diameter of ventral sucker (VS min), distance between the anterior end of the body and the ventral sucker (A-VS), distance between the oral sucker and the ventral sucker (OS-VS), distance between the ventral sucker and the union of the vitelline glands (VSVit), distance between the union of the vitelline glands and the posterior end of the body (Vit-P), distance between the ventral sucker and the posterior end of the body (VS-P), pharynx length (PhL), pharynx width (PhW), testicular length (TL), testicular width (TW), testicular perimeter (TP);
- Areas: body area (BA), oral sucker area (OSA), ventral sucker area (VSA), pharynx area (PhA), and testicle area (taking both testes together, TA);
- Ratios: body length over body width (BL/BW), body width at ovary level over cone width (BWOv/CW), oral sucker area over ventral sucker area (OSA/VSA), and body length over the distance between the ventral sucker and the posterior end of the body (BL/VS-P).

For the quantification of the body shape, the body roundness was measured (BR = BP^2^/4πBA). This measurement estimates how circular an object is, i.e., the expected perimeter of a circular object divided by the perimeter. Accordingly, a circular object has a roundness value of 1.0, whereas irregular objects show larger values [54,55].

A calibrated microscope was used for the measurements and images captured with a digital camera (Nikon Coolpix) were analyzed by CIAS (computer image analysis software) by means of the software Image-Pro Plus (version 5.0 for Windows, Media Cybernetics, Silver Spring, MD, USA).

#### 2.2.3. Numerical Methods

Morphological variation can be quantified by means of geometrical morphometrics [56]. The technique offers a size estimation in a way that different growth axes are integrated into a unique variable, i.e., the “centroid size” [57]. This single size-estimating variable reflects the variation in many directions, depending on the landmarks under study. The shape is defined by the relative positions after the correction for size, position and orientation. In that way, a freely available software allows for a more accurate quantification by conducting complex analyses, including significant biological and epidemiological features [58]. In morphometrics, statistical techniques allow for the testing of the null hypothesis of conspecific populations, i.e., if they are simply the allometric extension of each other provided a common allometric trend is identifiable [56,59,60].

Multivariate analyses were used to explore the morphometric data. With the aim of assessing between-samples morphometric variation, a size-free canonical discriminant analysis was applied on the covariance matrix of log-transformed measurements. This technique consists of removing the effect of within-group ontogenetic variation, regressing each character separately on the within-group first principal component, which is a multivariate estimate of size [61]. The analyses were carried out using BAC v.2 software [60,62,63]. Values were considered statistically significant when *p* < 0.05. Nonredundant measurements were used (i.e., one is not included in another one): BL, BW, BP, OS max, OS min, vs. max, vs. min, A-VS, VS-Vit, Vit-P, PhL, PhW and TL, where at least one dimension of the most important morphological structures was included. These remaining variables were all significantly correlated with the first principal component (PC1), which contributed 69% to overall variation. Therefore, PC1 could be considered as a general indicator of size [57]. Thus, the factor maps obtained (Figure 2) clearly and appropriately illustrate the differences in size when comparing the populations in question. The influence of within-group allometries was afterwards removed by using variables equivalent to an orthogonal projection of the data onto the first common principal component (i.e., all the common principal components except the first one). The resulting “allometry-free”, or size-free, variables were subsequently submitted to a canonical variate analysis (CVA), and Mahalanobis distances were derived [60,64].

Given that the size variation is consequently based on one variable only—namely the first common principal component—the univariate equivalent of Mahalanobis distance, which is also known as the Pearson’s “Coefficient of Racial Likeliness” [60], was applied for the estimation of its variation. The relationships of altitudinal variation with these distances were further statistically analyzed by nonparametric tests.

### 2.3. Genotyping Analyses

The molecular methods and techniques were applied to the aforementioned three Ecuadorian groups of liver flukes obtained in sheep and cattle from the zone of San Juan de Chillogallo, as well as to those specimens infecting cattle from the southern zone of Loja.

#### 2.3.1. DNA Markers

The sequence of the complete intergenic nuclear ribosomal DNA (rDNA) region, including the spacers ITS-2 and ITS-1 and the 5.8S gene, and the complete sequences of the two protein-coding genes *cox*1 and *nad*1 of the mitochondrial DNA (mtDNA) were selected to characterize the flukes. These molecular markers have already proved to be useful for the genetic characterization of *Fasciola* species and strains at local and regional levels [26,43] and in worldwide analyses [17], including the assessment of the spreading routes.

#### 2.3.2. DNA Sequencing

For DNA extraction, a small part of the anterior body region of fasciolids was individually processed. The methods therefore used have been previously described [43,65]. Materials were suspended in 400 μL of lysis buffer (10 mM Tris-HCl, pH 8.0, 100 mM EDTA, 100 mM NaCl, 1% sodium dodecyl sulfate SDS) containing 500 μg/mL Proteinase K (Promega, Madison, WI, USA). The digestion was performed for 2 h at 55 °C, including alternate shaking every 15 min. Methods previously outlined were followed concerning the procedure steps [26,43]. The phenol-chloroform extraction and ethanol precipitation method were applied for total DNA isolation. Each pellet was dried and resuspended in 30 μL sterile TE buffer (pH 8.0), and subsequently, this suspension was stored at −20 °C until needed.

Each DNA marker was amplified by PCR in an independent way for each liver fluke individual. Each PCR product was sequenced for a bona-fide haplotype characterization. Forward and reverse primers were designed in the regions flanking the rRNA genes 18S and 28S for the subsequent amplification of the complete ITS-1, 5.8S, ITS-2 region [28,65]. Forward and reverse primers designed in flanking regions allowed for the sequencing of the *cox*1 and *nad*1 genes in their complete length [17,26,43].

For the PCR amplification, the Biotools DNA polymerase^®^ (Biotools B&M Labs. S.A., Madrid, Spain) was used in a Verity-96 Well Thermal Cycler (Applied Biosystems, Thermo Fisher Scientific, Waltham, MA, USA). The program comprised one cycle of 2 min at 94 °C, 35 cycles of 1 min at 93 °C, 1 min at 55 °C and 1 min at 72 °C each, preceded by 2 min at 72 °C, and followed by a final cooling at 4 °C, for the ITS rDNA region, and one cycle of 1 min at 94 °C, 40–42 cycles of 1 min at 93 °C, 1 min at 52–55 °C and 2–3 min at 72 °C each, preceded by 5 min at 72 °C and followed by a final cooling at 4 °C, for the *cox*1 and *nad*1 mtDNA genes.

For the purification of the PCR product, the Ultra Clean™ PCR Clean-up DNA Purification System (MoBio, Solana Beach, CA, USA) was used following the manufacturer’s protocol and eluted in 50 μL of 10 mM TE buffer (pH 7.6). The final DNA concentration (in μg/mL) and the absorbance at 260/280 nm were determined in an Eppendorf BioPhotometer (Hamburg, Germany). Each molecular marker was sequenced on both strands by the dideoxy chain-termination method performed with the Taq dye-terminator chemistry kit on an Applied Biosystems 3730xl DNA Analyzer (Applied Biosystems, Foster City, CA, USA), by using the PCR primers

Sequence data from this article have been deposited in the GenBank Data Library under Accession Nos. MW867310–MW867323.

#### 2.3.3. Sequence Analyses

The software Sequencher v. 5.4.6 (Gene Codes Co., Ann Arbor, MI, USA) was used to edit and assemble the sequences, and the software CLUSTAL Omega [66] was used to align them by means of default parameters. Corresponding penalties for gaps were included in pairwise and multiple alignments. Total character differences were used to measure divergence of the sequences within and among different ITS-1 and ITS-2, *cox*1 and *nad*1 markers. All changes, comprising transitions (ts), transversions (tv) and insertions/deletions (indels), were considered as character states in MEGA v7.0 [67]. By means of the ALTER web server [68], the sequences aligned were collapsed to haplotypes, counting gaps as differences. Closely related sequences were searched by utilizing the BLASTN programme from the National Center for Biotechnology Information website (http://www.ncbi.nlm.nih.gov/BLAST) (accessed 15 December 2020). Comparative sequences were analyzed by comparing with available rDNA and mtDNA sequences of *F. gigantica, F. hepatica and Fasciola* spp. downloaded from the GenBank and also with the Valencia centre fasciolid haplotype collection.

#### 2.3.4. DNA Haplotype Nomenclature

The terminology to identify the haplotype (H) of the four aforementioned DNA markers follows the previously proposed combined haplotyping (CH) nomenclature [17,69]. According to this nomenclature, ITS-2 haplotypes are defined by numbers, and ITS-1 haplotypes by capital letters. Numbers are also utilized for the nucleotide and protein haplotypes of the mtDNA *cox*1 and *nad*1 genes. It is worth mentioning that haplotype codes are only definitive when the sequences are complete, i.e., full length sequences. When dealing with fragments or incomplete sequences, haplotype codes are considered only provisional.

## 3. Results

### 3.1. Morphometric Analyses of Sheep Liver Flukes

**Figure 3:**
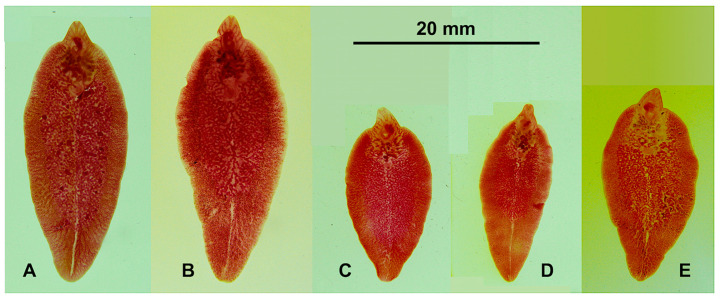
Standard liver fluke specimens of the populations infecting sheep in South America: (**A**,**B**) San Juan de Chillogallo, Ecuador (nEc); (**C**) Cajamarca, Peru (nCaj); (**D**) Mantaro valley, Peru (nMan); (**E**) Northern Altiplano, Bolivia (nAlt). All specimens shown at the same scale.

The morphometric values of South American populations of *F. hepatica* are noted in Table 1, with standard specimens shown in Figure 3. Values of *F. hepatica* from Spain and experimentally obtained *F. gigantica* from Egypt and Vietnam are shown in Table 2 and Table 3.

A general overlap is observed between populations of *F. hepatica* regardless of the geographical area of origin. The comparison of *F. hepatica* with *F. gigantica* also shows an overlap between the two species (including BR, BL/BW and VS-P).

However, it is worth mentioning the maximum and average values of the Ecuadorian fasciolid population, which are much higher than those of the other *F. hepatica* populations analyzed. The high values of the following parameters linked to size (BA, BL, BW, BWOv, BP, and BR) in the sheep specimens from Ecuador should be highlighted (Table 1) and compared with the same parameters in the *F. gigantica* populations from Egypt and Vietnam (Table 3).

A scatter plot of the first two principal components (PC) of the size variables shows that there are two differentiated zones along the horizontal axis (CP1) corresponding to the overlapping of the *F. hepatica* and *F. gigantica* populations. One zone is made up of geographical areas of Bolivia, Peru and Spain, where only *F. hepatica* is present. The other zone is made up of Egypt and Vietnam, corresponding to *F. gigantica* populations.

**Figure 4:**
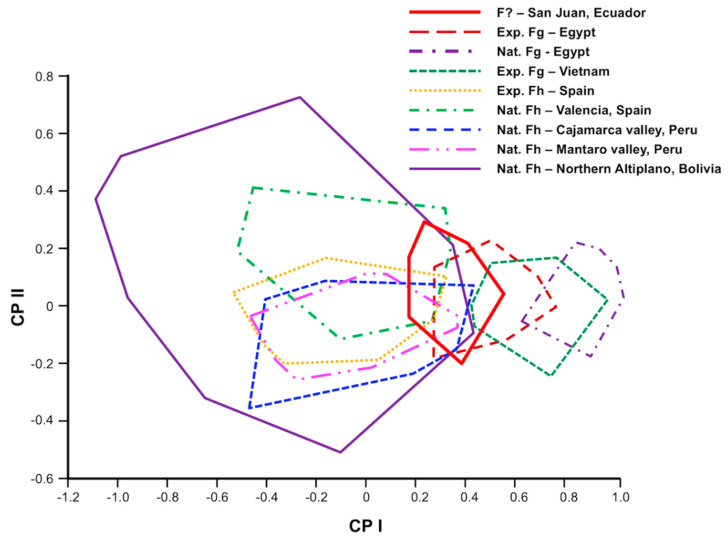
Factor map for the comparison of *Fasciola* specimens found naturally infecting sheep in San Juan in Ecuador (nEc), with *F. hepatica* specimens from Cajamarca (nCaj) and Mantaro (nMan) valleys in Peru, Northern Altiplano (nAlt) in Bolivia, and Valencia (nSp) in Spain, as well as with *F. hepatica* (Spanish isolate - expSp) and *F. gigantica* (Egyptian isolate - expEg, and Vietnamese isolate - expVi) from experimentally infected Guirra sheep. Samples are projected onto the first (PC1, 69%) and second (PC2, 11%) principal components. Each group is represented by its perimeter.

The resulting factor maps (Figure 4) illustrate global size differences in the populations analyzed. The *F. hepatica* populations from Peru (nCaj, nMan) and Spain (nSp) show a maximum and minimum size similar to that of the experimental *F. hepatica* standard population. The Bolivian population (nAlt) shares its maximum size with the other *F. hepatica* populations but presents a lower minimum size (Figure 4). The flukes from Ecuadorian sheep (nEc) have the biggest size recorded among *F. hepatica* populations. The experimental *F. gigantica* specimens from Egypt (expEg) included in the PCA (Figure 4) were 24 weeks old and show a clear separation from the experimental population of *F. hepatica* from Spain (expSp), which was also 24 weeks old. The factor maps show that, although in experimental populations the fluke size of *F. hepatica* and *F. gigantica* does not overlap (only a very little between expSp and expEg), the fluke size of experimental *F. gigantica* from Egypt (expEg) overlaps with the maximum size values of South-American *F. hepatica* populations (nCaj, nMan, nAlt). To avoid the effect of age, an experimental 52-week-old population from Vietnam (expVi) has been added to the comparison, showing a clear separation with all the *F. hepatica* populations excepting the population of Ecuador. To make the separation from *F. hepatica* even clearer, a natural population of *F. gigantica* found in slaughtered older sheep from Egypt (nEg) has additionally been included. The results show that the Ecuadorian samples (nEc) constitute the only population with a specimen size intermediate between that of *F. hepatica* populations (including both natural and experimental) and *F. gigantica* populations (also including both natural and experimental).

**Figure 5:**
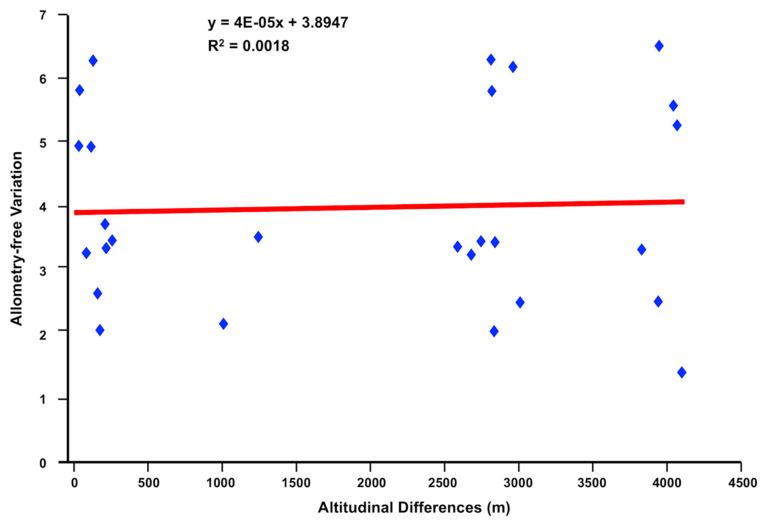
Plots of the pairwise Mahalanobis distances derived from allometry-free variables (vertical axis) against the corresponding altitudinal differences. Straight lines represent the linear regression prediction.

The size-free pattern of variation did not show any consistent relationship with altitudinal differences (Figure 5). Additionally, the presence of sperm in the seminal vesicle was microscopically confirmed in all the specimens found in sheep from Ecuador.

### 3.2. DNA Sequence Analyses

Two haplotypes of the complete ITS1-5.8S-ITS2 region were detected in the fasciolids infecting sheep and cattle in Ecuador. One haplotype was identical to the previously described *F. hepatica* haplotype Fh-1A (MG569980), including a 951 bp long intergenic region of 50.79% GC content. The second one includes a homozygous mutation in position 874 of the alignment and was described as Fh-2A (MG569978), with the same length and a 50.68% GC content. A variant of those two haplotypes was found and characterized by including a heterozygous mutation in the same 874 alignment position (Table 4). The most abundant in sheep and cattle in Ecuador proved to be the haplotype Fh-1A. The haplotype Fh-2A was detected only in cattle samples and the heterozygous variant (Fh-1/2A) only in some sheep samples.

ITS-1 proved to have the same sequence of 432 nucleotides and a 51.85% GC content in all specimens studied, corresponding to the haplotype Fh-HA of this spacer. The 5.8S gene was also very conserved in all specimens, with 154 base pairs and 53.25% GC. The 365-bp-long ITS-2 was the only one providing two haplotypes (Fh-H1, 48.49% GC; Fh-H2, 48.22% GC). There was only one single nucleotide polymorphism (SNP) in position 288 of the ITS-2 alignment, namely a “C” in Fh-H1, a “T” in Fh-H2, and “C/T” in the heterozygous variant (Fh1/2). This polymorphic position is not a position that differentiates between *F. hepatica* and *F. gigantica* (Table 4). The electropherograms of the rDNA intergenic sequence of all big-sized liver fluke specimens from sheep were thoroughly reviewed, especially in the positions that differentiate between *F. hepatica* and *F. gigantica*. No double peaks or ambiguous positions were detected, thus confirming its ascription to *F. hepatica*.

The mtDNA *cox*1 gene provided eight different sequences with the same length of 1533 bp and an average of AT content of 62.63%. Their alignment showed 15 variable positions (9 parsimony-informative and 6 singleton). Seven of these *cox*1 haplotypes are among the 69 reported previously in *F. hepatica*, corresponding to the haplotypes Fh*cox*1-16, Fh*cox*1-23, Fh*cox*1-53, Fh*cox*1-54, Fh*cox*1-55, Fh*cox*1-56, and Fh*cox*1-57. The eighth haplotype is a new one, found only in sheep, for which the code Fh*cox*1-70 has been assigned. Sheep and cattle samples only shared the haplotype Fh*cox*1-16. The COX1 protein was 511 aa long, with start/stop codons of ATG/TAG, and provided two haplotypes (Table 5). The difference between the two haplotypes was restricted to only one amino acid change in position 499 of the protein alignment (Table 5).

A comparative *cox*1 sequence analysis was performed with other complete length *F. hepatica* haplotypes, including Fh*cox*1-5, Fh*cox*1-16 and Fh*cox*1-42 from cattle and horses from Uruguay; haplotype M93388 from Salt Lake City, Utah, USA; and haplotype AF216697 corresponding to the Geelong strain from Australia. Their alignment was 1533 bp long and showed 30 nucleotide and 5 amino acid variable positions (Table 5).

The mtDNA *nad*1 gene provided six different sequences with the same length of 903 bp and an average AT content of 65.30%. Their alignment showed 6 variable positions (4 p-info and 2 singleton). All these six *nad*1 haplotypes are among the 51 *nad*1 haplotypes reported previously in *F. hepatica* and corresponding to the haplotypes Fh*nad*1-2, Fh*nad*1-6, Fh*nad*1-14, Fh*nad*1-23, Fh*nad*1-42 and Fh*nad*1-43. Sheep and cattle samples only shared the haplotype Fh*nad*1-14. The NAD1 protein showed only one 300-aa-long haplotype with start/stop codons of GTG/TAG in all specimens analyzed (Table 6).

Comparative *nad*1 alignment analyses were performed with other complete length *F. hepatica* haplotypes, including Fh*nad*1-2, and Fh*nad*1-12 and Fh*nad*1-14 reported from cattle and horses in Uruguay; haplotype M93388 from Salt Lake City, Utah, USA; and haplotype AF216697 corresponding to the Geelong strain from Australia. Their alignment was 903 bp long and contained 12 nucleotide and 1 amino acid variable positions that corresponded to the intraspecific variability reported for *F. hepatica* (Table 6).

Similarly, as done with the rDNA sequences, the electropherograms of the mtDNA *cox*1 and *nad*1 of all big-sized liver fluke specimens from sheep were carefully checked, especially in the positions that differentiate between *F. hepatica* and *F. gigantica*. Neither introgression fragments nor heteroplasmic positions were found, thus confirming its ascription to *F. hepatica*.

The distribution of the haplotypes of each of the aforementioned rDNA and mtDNA markers analyzed according to localities surveyed in Ecuador, and their previous findings in other countries are noted in Table 7.

## 4. Discussion

### 4.1. Phenotypic Analysis

#### 4.1.1. *Fasciola hepatica* from Highland and Lowland Sheep

The geographical origins of the liver flukes from naturally infected sheep analyzed in this study include highlands and lowlands. Quantitative morphological variation informs about both genetic variation and external influences [70]. High-altitude environment factors exert an influence on mammals, so those born and living at high altitude show morphological and physiological characteristics different from those of mammals inhabiting low altitudes [71,72].

Although the *F. hepatica* population from the Bolivian Altiplano shows a wider size range, reaching uterus gravidity at a lower size (Figure 5) [73], this Altiplanic pattern differing from the valley pattern of the Peruvian Cajamarca and Mantaro [74], the results of the present study show that there is no apparent relationship between the shape of fasciolid adults with respect to the difference in altitude or geographical location.

Using an allometric model, the definitive host species proved to decisively influence the size of *F. hepatica* adults, but these influences do not persist in a subsequently infected rodent-definitive host model [46]. In natural populations, only slight differences were found in allometric models (BL, BW, P vs. BA or BL) between *F. hepatica* adults from highland and low-land populations of Bolivian and Spanish sheep [75]. Nevertheless, liver fluke populations from Altiplanic sheep and cattle, which have proved to be efficient reservoirs in the very high-altitude areas [76], proved to have a smaller uterus area than European lowland populations in the same host species [73]. Oxygen is needed for the production of eggs in *F. hepatica* [77,78,79]. Thus, hypoxia from which vertebrate hosts living in high altitude areas suffer may lead to a reduced egg production in trematodes. Moreover, although trematode uteri are traditionally not considered to be storage organs, the size of the uterus is primarily adapted for the egg to reach maturity along the uterine journey. In the Northern Bolivian Altiplano, climatic conditions, freshwater characteristics and ecological requirements of lymnaeids allow for fascioliasis transmission throughout the entire year [23,80]. In the Altiplanic pattern, egg storage is not essential, and this explains why smaller sizes are detected, in contrast to the valley pattern where fascioliasis presents a typical seasonal transmission just as in the northern Hemisphere.

Allometry-free shape appears as a more stable trait than size in fasciolid species [74]. This is in agreement with observations made on insect vectors of *Trypanosoma cruzi* (Triatominae), in which allometry-free shape may require important external changes to be significantly modified [58].

#### 4.1.2. Liver Flukes from Ecuadorian Sheep Compared to *F. hepatica* and *F. gigantica*

The development of *F. hepatica* and *F. gigantica* in the definitive host [81,82] follows growth curves according to a logistic model, in which the morphometric development of the adult stage is “damped” and cannot exceed certain characteristic maximums of Ym. This logistic model of the body growth and development is characterized by two phases [49,82]: (i) the “exponential phase” of the logistic growth corresponds to the body development during the migration in the abdominal cavity and liver parenchyma and subsequent development and sexual maturation in the biliary duct system up to the onset of egg production; (ii) the “saturated phase“ of logistic growth starts from this moment and leads to a gradually stationary growth after oviposition.

In areas of countries of Africa and Asia where the two species of *Fasciola* overlap thanks to the coexistence of the respective specific lymnaeid species, the comparative multivariant analyses show flukes of a size intermediate between *F. hepatica* and *F. gigantica*, as demonstrated in Egypt [53], Iran [83], Pakistan [84] and Bangladesh [85].

The high maximum and mean values of all size-linked parameters in the Ecuadorian liver flukes from sheep are unexpected and should undoubtedly underlie, and justify, previous classifications of fasciolids in this country as *F. gigantica*, whether unpublished or published [41]. Indeed, size has traditionally been the main and even only characteristic considered in most of the past fasciolid species classifications in livestock, which led to several misclassifications later verified [26].

In cattle, a study showed that BL/BW, BR and VS-P are useful tools for differentiating between “genetically pure” *F. hepatica* from southern Europe and “genetically pure” *F. gigantica* from Burkina Faso [52]. In sheep, the results of the present study show an overlap in each of these three markers between *F. hepatica* and *F. gigantica*, although mean values are clearly different (compare Table 1, Table 2 and Table 3). Regarding the flukes from Ecuadorian sheep, VS-P appears to be a parameter showing evident intermediate characteristics which supports the conclusion of the phenotypic results on fluke size.

### 4.2. Molecular Analysis

#### 4.2.1. DNA Sequence Characterization of Fasciolids from Ecuador

Big-sized fasciolid flukes found in sheep from Ecuador molecularly prove to belong to *F. hepatica* and all haplotypes of the four DNA markers used fall within the intraspecific variability reported for this species in European and American countries [17]. The same conclusion is reached in the haplotyping of the liver flukes collected in cattle from the three selected localities surveyed, one close to Quito and the other two in the southern zone neighboring the Peru border.

ITS-1 and ITS-2 are evolutionarily conserved markers very useful for species differentiation. Their consequent low variability at the local level does therefore provide little additional information about population genetics because it has been observed in invertebrates in general unless microsatellites and/or minisatellites varying in repeat numbers are included in their sequences [86]. In those regions of the world where only *F. hepatica* is present, and consequently there is no possibility for hybridization with *F. gigantica*, only FhITS1-HA is known [17], as observed in Ecuador in flukes from both sheep and cattle. Regarding ITS-2, the finding of the haplotype FhITS2-H1 in both sheep and cattle and in all localities surveyed agrees with the wide distribution of this haplotype. Similarly, it may be argued with the generally more geographically scarcely distributed FhITS2-H2. The detection of heterozygotic characteristics in the only variable position differentiating these two ITS-2 haplotypes (Table 4) speaks about a very recent mating or “hybridization” of parental flukes harboring one and the other haplotypes. This, in its turn, suggests usual mixing of populations coming from different parts of Ecuador in the area of San Juan for the daily supply needed for a highly populated city as Quito.

The faster evolutionary rate of the mtDNA genome underlies the wider haplotype variability of the *cox*1 and *nad*1 genes [86]. In *Fasciola*, the worldwide geographical spread of different haplotypes of these mtDNA genes is linked to man-made livestock movements occurred along the post-domestication period during the last 12,000–10,000 years [17], and in the Americas after European livestock introduction by the Spanish “conquistadores” and subsequent intracontinental spread during only the last 500 years [43]. The scattered distribution of the mutations throughout the whole length in these two long genes is worth mentioning (Table 5 and Table 6). This indicates that valuable information is lost when only using short fragments of these genes in analyses of fasciolid flukes. The assessment of past migrating routes may be misinterpreted or the correct overview may not be reached when only using short fragments of mtDNA genes, as it may happen with liver flukes in Ecuador [87]. Indeed, the difficulties in correctly assessing the spreading routes of fasciolid flukes become evident when considering the overall mixing of fluke populations as a consequence of livestock movements driven by humans, inside countries and between countries as in South America [43], but also between continents [17,88]. Additionally, this means that for a correct assessment of the usefulness of DNA markers for the differentiation between *F. hepatica* and *F. gigantica*, appropriate fasciolid populations should be selected; i.e., comparing fasciolid specimens from Ecuador with fasciolids from countries of Southeastern and Far East Asia [89], where the two fasciolid species overlap and hybrid fasciolids are widely distributed, does not furnish the adequate baseline for such a purpose.

The detection of eight haplotypes in *cox*1 and six haplotypes in *nad*1 indicates high genetic variability of *F. hepatica* in Ecuador. However, in *cox*1, only one nucleotide codon gives rise to an amino acid change (Table 5), whereas in *nad*1, all mutations prove to be silent (Table 6). This high haplotype mtDNA variability agrees with the heterogeneous origin of the livestock nowadays present in Ecuador. The present mix of *F. hepatica* haplotypes in Ecuador is the result of successive livestock introductions from abroad during the following two periods:

- The colonial period: Three introduction routes were involved. (i) The maritime route through the Pacific coast was the initial entry for livestock from Central America, at the beginning of the colonization of South America. (ii) The southern terrestrial route from Peru started early thereafter, as soon as the European colonizers learned about the interest of Quito for the old Incas. (iii) The northern route through the border with present day Colombia was implemented later, with an exchange of silver transport from the Bolivian Potosi mine up to the Venezuelan haven of Cartagena. The latter silver transport route might have been the most important concerning the probability of introduction of *F. hepatica* haplotypes from both southern and northern countries into Ecuador.
- The post-colonial period: The original European livestock evidenced problems of adaptation to the Ecuadorian environment both throughout the tropical lowlands along the coast as well as in the Andean highlands. During the XIXth and XXth centuries, Ecuador became a great livestock importer, mainly to improve the local cattle breeds. Importations of many thousands of animals were performed from the XIXth century, but mainly during the first two thirds of the XXth century. These importations were mainly from other countries of South America, but sporadically also from Europe and Asia, as well as North and Central America [90].

These livestock introductions from abroad underlie the wide diversity of haplotypes detected in Ecuadorian sheep and cattle, given the capacity of metacercariae to infect different livestock species [47,91]. Moreover, fasciolid infection does not induce premunition, so re-infections lead to fluke accumulation within the same host individual [92,93]. This means that livestock importation by terrestrial herd movements from one country to another and through other countries located in between may easily become the source of the introduction of more than one haplotype, and that introduced haplotypes may finally infect other livestock species in situ once in the new country. The detection of five *cox*1 haplotypes and three *nad*1 haplotypes suggests the mix of different lineages in the big-sized *F. hepatica* specimens in sheep (Table 7).

Haplotypes such as Fh*cox*1-16 and Fh*cox*1-23—but also Fh*cox*1-53 and Fh*cox*1-55, as well as Fh*nad*1-2, Fh*nad*1-14 and Fh*nad*1-23, all of them shared by Ecuador with many other countries of South America or even Mexico (Table 7)—suggest introductions by livestock exchange inside the Americas. The haplotypes Fh*nad*1-2 and Fh*nad*1-6 are shared by Ecuador with Spain and may be remains of introductions from Europe during the colonial period. The haplotypes Fh*cox*1-54, Fh*cox*1-56, Fh*cox*1-57, and the new Fh*cox*1-70, as well as Fh*nad*1-42 and Fh*nad*1-43, have so far never been found in other countries. Research today underway shall clarify whether they are also present in other countries.

The pronouncedly higher number of different haplotypes in San Juan when compared to the haplotype number in southern Peru (Table 7) also indicates a mixing of populations from different parts of Ecuador in the area of San Juan for the regular demand of the big city of Quito. Only Fh*cox*1-16 and Fh*nad*1-14 have been found in both San Juan and the southern Ecuador border, which suggests that livestock is nowadays not transported to Quito from so far inside the country.

#### 4.2.2. American Big-Sized *F. hepatica* Introduction Routes

The *F. hepatica* worms here described from sheep in Ecuador share the characteristic of an abnormally big size with several liver flukes reported from another American country, namely USA.

In North America, zebu cattle and buffaloes were imported from India and probably also from Africa along the colonization period starting 500 years ago [94,95,96]. Importations from India into the Gulf coast occurred in 1875 and 1906 and may explain the presence of three different types of *Fasciola* in the USA [36]: in several states, they are identical to *F. hepatica*, those from Texas and Florida approach *F. gigantica*, and those from the Gulf Coast area show intermediate form characteristics. *Fasciola halli* described in Texas and Louisiana and *F. californica* in California [97] were probably intermediate forms resulting from introgressions of imported *F. gigantica* into USA-native *F. hepatica* [17]. Although *F. gigantica* was unable to adapt due to *Radix* species unavailability in USA, cross-breeding could have occurred within the livers of the initially imported animals (at that time, directly released into the field without prior quarantine, as in many developing countries today), enabling the foreign *F. gigantica* to encounter native *F. hepatica*, with subsequent DNA introgression. Hybrids unable to develop in USA-native snails subsequently disappeared, but others retained viability due to their capacity to use US-native lymnaeids [17].

In Ecuador, three questions are posed to understand such *F. gigantica*-sized flukes in this country: from where, when and how were *F. gigantica* or *F. gigantica*-like flukes introduced to subsequently give rise to such a local big sized strain.

The first sheep (criollo breeds) were introduced by the “Spanish conquistadores” already from the beginning of the colonization period. By the XVIth century, sheep were already widely spread in the Pichincha province [98]. After the long colonization period, sheep importations were also made but at a lesser level than with cattle because of the lower interest in sheep breed improving [99]. The origins of these sheep importations were initially Europe, and later Australia, New Zealand and USA in the 1964–1987 period [100]. Consequently, only those from southern States of the USA could have been risky if infected by the big-sized (*F. gigantica*-like) liver flukes recorded in the USA [36] and which could be adapted to be transmitted by North American non-*Radix* lymnaeids, given the absence of *F. gigantica* in Europe and Oceania

The first cattle arrived in Ecuador on boats from Nicaragua in 1537. Most of the numerous livestock importations during the XIX and XX centuries concerned cattle from different continents except Africa [90]. Most cattle importations were from other South American countries and have been implemented even very recently, as the importation of 12,000 bovines from Paraguay in the 2015–2017 period [101]. Of special interest was the importation of cattle of the Sahiwal breed from Pakistan through the Ecuadorian coast in 1974 [90]. This breed adapted very well to the Ecuadorian tropics. *Fasciola gigantica* is widely spread throughout most of Pakistan [21], where it has been seen to reach big size in buffaloes [84]. If these imported Pakistani animals were infected by *F. gigantica*—which is highly probable considering its very high prevalence in this Asian country [21]—their release in the environment of Ecuador might have offered their additional infection by local *F. hepatica* and subsequent hybridization giving rise to intermediate forms. Those hybrid flukes which succeeded to use original Ecuadorian lymnaeids for their transmission might have been the origin of the big sized liver fluke lineage having reached the present in Ecuador.

Worth mentioning also is the importation of Brown Swiss cattle from Ohio and Minnesota, USA, to Santo Domingo de los Tsáchilas in 1986. Although *F. gigantica*-like flukes or intermediate forms were not present in these northern states of the USA [36], the 132 cows imported were transported with trucks along 3000 km up to Miami along a three-day journey, allowing the animals to go out and freely feed locally in the different states every 12 h. Finally, the cows were transported by a direct flight from Miami to Ecuador [90]. Hence, the infection of these cows by *F. gigantica*-like flukes or intermediate forms could have occurred once in the southern states of the USA [36]. In that way, large-sized liver flukes could have been introduced in the area where reported big-sized *F. hepatica* infecting sheep have been found in Ecuador. Indeed, the old route from Quito to the Pacific Ocean covered the westward transect Quito–San Juan–Chiriboga–Santo Domingo de los Tsáchilas from 1888. All transport from the different western coastal localities to Quito was made through several routes which converged in Santo Domingo de los Tsáchilas [102].

### 4.3. Radix Absence and the Evolutionary Snail Vector Filter

Although the aforementioned date of 1974 as the most probable for a *F. gigantica* introduction with cattle from Pakistan [90] may be considered very recent, the time elapsed is more than sufficient to explain the absence of heterozygotic positions with *F. gigantica* in the two rDNA spacers ITS-2 and ITS-1 and the lack of detection of introgressed sequences or heteroplasmic positions in the complete mtDNA sequences of the *cox*1 and *nad*1 genes in the large-sized *F. hepatica* flukes from Ecuadorian sheep. In the case of the 1986 cow importation from USA [90], if liver fluke intermediate forms were introduced into Ecuador, these would have previously been originated in southern USA [36] by an adaptation to North American lymnaeid species of the *Galba*/*Fossaria* group such as *L. humilis* or *L. bulimoides* [103] and subsequently successfully established in Ecuador. Indeed, the concerted evolution mechanisms acting on the rDNA operon may reach sequence homogenization quite rapidly from the timely perspective [104]. Similarly in the case of mtDNA, heteroplasmy longevity until return to homoplasmy has also been observed to occur quite rapidly, from just a few generations in cattle up to 500 generations in the shorter life-span insects [105].

In studies on the epigenetic phenomenon of nucleolar dominance in hybrid organisms, it has been shown that the rRNA genes of one parental species may become transcriptionally dominant over the rRNA genes of the other parental species [104]. In fasciolid liver flukes, the specificity regarding different lymnaeid vector species should undoubtedly play a crucial role in such a phenomenon. The availability of given lymnaeid species and the asexual clonal intramolluscan larval development should act as a natural selection filter. In that way, the capacity for sexual crossbreeding of these hermaphroditic flukes and maintain a hybrid characteristic lineage becomes greatly reduced by the increasingly low probability of encountering other needed hybrids inside the liver of the same host individual, because of non-local-snail adapted hybrid elimination by the lymnaeid vector filter.

Such an evolutionary filter at vector level should additionally accelerate the elimination of hybrid genetic characteristics. In the Americas, *F. gigantica* lymnaeid vector species of the Old-World *Radix* group are absent. Only in the USA has a *Radix* species been found, namely introduced *Radix auricularia*, although it is mostly confined to artificial water collections in man-made environments and molecularly similar to the same European species [103]. In South America, a radicine lymnaeid has never been found. This absence of *Radix* lymnaeids explain the lack of capacity of *F. gigantica* to colonize the New World, even despite human activities having involuntarily offered occasions for such an introduction. Opposite to this, the anthropogenic introduction of *F. hepatica* was successful because of its subsequent adaptation to American native *Galba*/*Fossaria* lymnaeid species, additionally to the introduction of the European *Galba truncatula* [17].

In Ecuador, only five lymnaeid species have been reported, namely the more geographically restricted *Pseudosuccinea columella* [106,107] and four more widely spread species of the *Galba*/*Fossaria* lymnaeid group including *Lymnaea cousini* [108], *L. schirazensis* [109], *L. cubensis* [110] and *L. neotropica* [42]. All these species are *F. hepatica* vectors, except *L. schirazensis* which is a nonvector species [109,111]. Thus, *F. gigantica* from Pakistan could not found an appropriate scenario for a successful introduction.

The large size of the flukes found in the USA and Ecuador poses the question about why these fasciolids do not return to the normal *F. hepatica* size and keep the large phenotype at least in the mid-term of several decades. In fasciolids, adult stage phenotype has been observed to be linked to rDNA but not always to mtDNA [17]. Unequal crossing over, high-frequency gene conversion, and large deletion underlying recombination mechanisms driving the rapid concerted evolution of the rDNA tandem repeats are known to efficiently homogenize the 45S rDNA single precursor transcribing the rDNA genes (18S, 5.8S and 28S) and spacers (ITS-1, ITS-2 and IGS) sequences by, in essence, counteracting mutation effects [112]. However, concerted evolution does not act on two aspects with substantial ecological and evolutionary consequences such as the operon copy number and the length of the intergenic spacer IGS [104]. Indeed, unequal crossover plays an important role in the generation of such two heterogeneities, and rRNA transcription is directly related to the growth and development of the organisms. A higher number of rRNA units provides a higher transcription capacity enabling for increasing growth [104]. Hybridization of *F. hepatica* and *F. gigantica* may give rise to a number of rRNA unit copies higher than usual in genetically pure *F. hepatica*, thus allowing for bigger size development. Natural selection, including lymnaeid vector species specificity and local availability as the main effectors, may further contribute to maintain balanced rDNA dosage across unlinked rDNA arrays, as seen in other organisms [113]. This situation of a timely stable evolutionary bottleneck at the snail vector level always acting in the same sense is the opposite to what happens in areas of Africa and Asia, where the local coexistence of *Galba*/*Fossaria* vector species with *Radix* vector species in the same transmission focus offers daily alternating development filtering towards *F. hepatica* and *F. gigantica* to an evolving lineage, or zonal overlapping presence of *Galba*/*Fossaria* and *Radix* vector species offering similar alternating filtering possibilities but at seasonal scale (as e.g., in altitudinal transhumance) [17]. In the ten-chromosome-pair karyotype of *F. hepatica*, rDNA is located in the short arms of the fifth homologous pair [114].

## 5. Conclusions

Two important public health aspects may be concluded from the results obtained. First, the more pathogenic *F. gigantica* does not appear to be able to colonize the New World. Even if inadvertently introduced with imported livestock from Africa or Asia, the absence of *Radix* snail species did not allow for normal transmission, and the potential hybridization with local American *F. hepatica* would progressively lead the consequent lineage to a genetic uniformization towards *F. hepatica* in a relatively short period. This does not mean, however, that it should be taken into account that pathogenicity is also linked to fluke size and that the intermediate hybrid forms are pronouncedly bigger than normal *F. hepatica*.

Second, it is evident that transborder liver fluke introductions occur due to the livestock importations from other countries. The risk of transborder spread of the resistance to triclabendazole should be consequently considered. Livestock treatments should be therefore applied at the level of the exporting country and appropriate quarantine and specific diagnostics applied in the importation country immediately after livestock arrival.

## Figures and Tables

**Table 1:** Comparative morphometric data (extreme values, mean and standard deviation) of natural liver flukes collected in highland sheep from the Northern Altiplano in Bolivia, Mantaro valley and Cajamarca valley in Peru, and San Juan in Ecuador (*n* = number of individuals).

Adult Measurements (mm)	Altiplano Bolivia *n* = 201 nAlt	Mantaro Peru *n* = 47 nMan	Cajamarca Peru *n* = 130 nCaj	San Juan Ecuador *n* = 42 nEc
Body area, BA	31.11–236.14	79.89–250.58	47.34–283.95	185,21–352.28
	106.39 ± 3.35	149.30 ± 5.64	135.87 ± 3.73	266.88 ± 4.78
Body length, BL	9.64–31.04	13.41–27.75	13.48–30.97	21.49–32.78
	18.08 ± 0.31	19.70 ± 0.40	18.86 ± 0.31	28.38 ± 0.41
Body width, BW	4.23–13.41	7.60–13.93	5.06–14.23	11.74–15.25
	8.26 ± 0.14	10.88 ± 0.23	10.25 ± 0.14	13.31 ± 0.14
BL/BW ratio	1.41–3.74	1.30–2.46	1.31–3.73	1.68–2.62
	2.22 ± 0.03	1.83 ± 0.04	1.86 ± 0.03	2.14 ± 0.04
BW at ovary level, BWOv	3.51–11.71	5.48–11.31	4.23–11.97	8.20–11.63
	6.71 ± 0.12	8.50 ± 0.19	8.30 ± 0.11	9.54 ± 0.12
Body perimeter, BP	26.89–68.35	33.71–64.51	30.20–71.11	44.36–77.30
	47.51 ± 0.72	48.15 ± 0.98	45.70 ± 0.66	65.84 ± 0.92
Body roundness, BR	1.11–2.12	1.13–1.48	1.09–1.94	1.16–1.54
	1.53 ± 0.01	1.26 ± 0.01	1.25 ± 0.01	1.33 ± 0.01
Cone length, CL	1.32–3.04	1.36–2.59	1.32–2.98	1.88–2.81
	2.12 ± 0.02	1.89 ± 0.04	2.11 ± 0.02	2.26 ± 0.03
Cone width, CW	1.78–3.92	2.30–4.21	2.12–4.41	2.77–4.44
	2.65 ± 0.03	3.04 ± 0.06	3.17 ± 0.04	3.54 ± 0.06
BWOv/CW ratio	1.53–3.83	1.86–3.94	1.79–3.72	2.19–4.19
	2.53 ± 0.03	2.82 ± 0.07	2.64 ± 0.03	2.72 ± 0.06
Oral sucker area, OSA	0.21–0.66	0.19–0.72	0.20–0.67	0.29–0.66
	0.38 ± 0.01	0.42 ± 0.01	0.46 ± 0.01	0.49 ± 0.01
Maximum diameter of the oral sucker, OSmax	0.53–1.06	0.61–1.02	0.63–1.14	0.79–0.98
	0.76 ± 0.01	0.81 ± 0.01	0.84 ± 0.01	0.88 ± 0.01
Minimum diameter of the oral sucker, OSmin	0.42–0.86 0.63 ± 0.01	0.29–0.90 0.62 ± 0.02	0.36–0.85 0.66 ± 0.01	0.35–0.88 0.67 ± 0.02
Ventral sucker area, VSA	0.44–1.23	0.49–1.26	0.53–1.22	0.82–1.20
	0.78 ± 0.01	0.82 ± 0.02	0.89 ± 0.01	1.01 ± 0.02
Maximum diameter of the ventral sucker, VSmax	0.75–1.25	0.83–1.31	0.89–1.29	1.07–1.35
	1.00 ± 0.01	1.08 ± 0.01	1.12 ± 0.01	1.18 ± 0.01
Minimum diameter of the ventral sucker, VSmin	0.67–1.29	0.72–1.22	0.77–1.21	0.95–1.19
	0.98 ± 0.01	0.95 ± 0.01	0.99 ± 0.01	1.07 ± 0.01
OSA/VSA ratio	0.31–0.71	0.31–0.69	0.23–0.76	0.28–0.64
	0.49 ± 0.01	0.51 ± 0.01	0.52 ± 0.01	0.49 ± 0.01
Distance between the anterior body end and ventral sucker, A-VS	1.52–3.35	1.75–2.88	1.61–3.26	2.19–3.22
	2.24 ± 0.02	2.29 ± 0.04	2.48 ± 0.02	2.82 ± 0.03
Distance between oral sucker and ventral sucker, OS-VS	0.87–2.56	1.16–2.15	1.19–2.40	1.68–2.52
	1.60 ± 0.02	1.66 ± 0.03	1.81 ± 0.02	2.13 ± 0.03
Distance between ventral sucker and vitelline gland union, VS-Vit	4.49–19.82	8.11–17.36	6.78–20.60	14.14–23.60
	11.32 ± 0.22	12.30 ± 0.30	11.56 ± 0.21	19.40 ± 0.42
Distance between vitelline gland union and posterior body end, Vit-P	0.49–9.83	2.64–8.09	2.69–9.47	3.65–10.44
	3.76 ± 0.14	5.20 ± 0.19	4.91 ± 0.12	6.56 ± 0.23
Distance between ventral sucker and posterior body end, VS-P	7.11–27.39	11.27–25.36	11.39–28.37	18.78–30.47
	15.07 ± 0.31	17.50 ± 0.43	16.47 ± 0.30	25.96 ± 0.43
BL/VS-P ratio	0.88–1.42	1.09–1.19	1.08–1.22	1.06–1.19
	1.22 ± 0.01	1.13 ± 0.004	1.15 ± 0.003	1.09 ± 0.004
Pharynx area, PhA	0.05–0.34	0.13–0.31	0.12–0.35	0.11–0.30
	0.17 ± 0.003	0.20 ± 0.01	0.21 ± 0.004	0.20 ± 0.01
Pharynx length, PhL	0.37–0.93	0.58–0.91	0.55–0.96	0.60–1.06
	0.68 ± 0.01	0.75 ± 0.01	0.78 ± 0.01	0.85 ± 0.02
Pharynx width, PhW	0.18–0.50	0.25–0.48	0.24–0.55	0.52–0.19
	0.34 ± 0.004	0.36 ± 0.01	0.36 ± 0.005	0.33 ± 0.01
Testes area, TA	9.31–89.48	22.54–71.71	13.10–112.85	57.36–121.39
	37.43 ± 1.35	48.33 ± 1.92	46.25 ± 1.32	96.11 ± 2.22
Testes length, TL	3.12–14.62	5.56–12.70	4.54–23.53	7.85–18.77
	8.12 ± 0.17	9.05 ± 0.26	8.86 ± 0.20	14.06 ± 0.35
Testes width, TW	2.84–8.97	4.84–9.87	3.12–9.07	7.69–10.33
	5.69 ± 0.10	7.01 ± 0.16	6.84 ± 0.09	8.93 ± 0.10
Testes perimeter, TP	13.06–48.53	20.08–41.44	16.76–48.75	33.35–51.66
	26.94 ± 0.56	30.68 ± 0.75	28.71 ± 0.44	44.55 ± 0.60
Testes roundness, TR	1.26–2.50	1.22–2.07	1.19–1.94	1.36–1.99
	1.57 ± 0.01	1.58 ± 0.03	1.45 ± 0.01	1.66 ± 0.02

**Table 2:** Comparative morphometric data (extreme values, mean and standard deviation) of natural and experimental liver flukes of *Fasciola hepatica* from lowland sheep (*n* = number of individuals).

Adult Measurements (mm)	Experimental Spanish Isolate *n* = 127 expSp		Natural, Valencia, Spain *n* = 37 nSp	
	Extreme Values	Mean ± SD	Extreme Values	Mean ± SD
Body area, BA	68.09–227.11	129.78 ± 3.17	75.09–239.13	142.75 ± 6.22
Body length, BL	12.45–26.68	18.52 ± 0.29	14.21–31.17	20.82 ± 0.64
Body width, BW	6.88–12.74	10.19 ± 0.10	7.49–12.76	9.75 ± 0.16
BL/BW ratio	1.30–2.62	1.82 ± 0.02	1.70–2.89	2.14 ± 0.05
BW at ovary level, BWOv	5.69–10.17	7.98 ± 0.08	6.45–10.57	8.13 ± 0.17
Body perimeter, BP	33.22–66.06	47.91 ± 0.64	39.85–71.41	52.90 ± 1.33
Body roundness, BR	1.23–1.73	1.43 ± 0.01	1.31–1.76	1.46 ± 0.02
Cone length, CL	1.10–3.07	2.01 ± 0,03	1.55–2.98	2.10 ± 0.06
Cone width, CW	2.08–4.19	3.27 ± 0.03	2.46–4.12	3.25 ± 0.06
BWOv/CW ratio	1.86–3.81	2.46 ± 0.03	2.03–3.90	2.51 ± 0–05
Oral sucker area, OSA	0.24–0.53	0.39 ± 0.01	0.33–0.66	0.44 ± 0.01
Maximum diameter of the oral sucker, OSmax	0.70–1.01	0.86 ± 0.01	0.63–1.00	0.84 ± 0.01
Minimum diameter of the oral sucker, OSmin	0.33–0.74	0.57 ± 0.1	0.52–0.86	0.67 ± 0.01
Ventral sucker area, VSA	0.56–1.31	0.98 ± 0.01	0.95–1.35	1.13 ± 0.01
Maximum diameter of the ventral sucker, VSmax	0.89–1.40	1.19 ± 0.01	0.92–1.40	1.19 ± 0.02
Minimum diameter of the ventral sucker, VSmin	0.79–1.23	1.04 ± 0.01	0.68–1.48	1.06 ± 0.03
OSA/VSA ratio	0.24–0.58	0.41 ± 0.01	0.27–0.51	0.39 ± 0.01
Distance between anterior body end and ventral sucker, A-VS	1.16–3.09	2.13 ± 0.03	1.66–3.15	2.32 ± 0.05
Distance between oral sucker and ventral sucker, OS-VS	0.64–2.50	1.56 ± 0.03	1.13–2.49	1.65 ± 0.05
Distance between ventral sucker and vitelline gland union, VS-Vit	7.71–19.75	12.33 ± 0.23	9.65–22.97	13.84 ± 0.47
Distance between vitelline gland union and posterior body end, Vit-P	1.99–7.02	4.35 ± 0.09	1.12–6.89	3.45 ± 0.20
Distance between ventral sucker and posterior body end, VS-P	10.43–24.66	16.68 ± 0.29	11.21–26.84	17.29 ± 0.58
BL/VS-P ratio	1.04–1.20	1.11 ± 0.002	1.15–1.27	1.21 ± 0.004
Pharynx area, PhA	0.11–0.34	0.20 ± 0.003	0.15–0.31	0.22 ± 0.01
Pharynx length, PhL	0.51–1.07	0.77 ± 0.01	0.59–0.89	0.74 ± 0.01
Pharynx width, PhW	0.26–0.56	0.36 ± 0.004	0.30–0.51	0.40 ± 0.01
Testes area, TA	24.12–89.10	50.99 ± 1.29	29.90–87.37	46.27 ± 2.35
Testes length, TL	5.33–14.50	8.92 ± 0.17	6.03–14.53	9.39 ± 0.32
Testes width, TW	4.66–9.16	7.37 ± 0.07	5.08–9.38	6.42 ± 0.16
Testes perimeter, TP	22.88–47.93	33.13 ± 0.49	20.81–44.36	30.93 ± 0.82
Testes roundness, TR	1.41–2.25	1.74 ± 0.02	1.30–2.22	1.69 ± 0.04

**Table 3:** Comparative morphometric data (extreme values, mean and standard deviation) of natural and experimental liver flukes of *Fasciola gigantica* from lowland sheep (*n* = number of individuals).

Adult Measurements (mm)	Experimental Egyptian Isolate *n* = 100 expEg	Natural Giza, Egypt *n* = 31 nEg	Experimental Vietnam Isolate *n* = 70 expVi
Body area, BA	156.61–305.35	385.29–556.58	438.46–474.11
	219.64 ± 2.87	470.0 ± 26.35	460.97 ± 9.58
Body length, BL	24.41–40.51	40.10–54.98	26.88–46.85
	31.47 ± 0.30	48.39 ± 3.36	36.17 ± 4.05
Body width, BW	8.28–12.04	8.73–12.45	9.82–16.30
	9.97 ± 0.09	10.63 ± 0.91	12.89 ± 1.27
BL/BW ratio	2.23–4.31	3.59–5.45	1.84–4.59
	3.18 ± 0.04	4.58 ± 0.47	2.83 ± 0.41
BW at ovary level, BWOv	6.33–9.98	9.05–11.53	6.33–9.98
	8.06 ± 0.07	10.07 1.02	8.06 ± 0.07
Body perimeter, BP	57.98–105.24	57.63–114.17	96.70–104.11
	74.41 ± 0.07	101.88 ± 10.32	100.10 ± 3.73
Body roundness, BR	1.53–3.47	2.11–2.48	1.67–1.81
	2.02 ± 0.02	2.28 ± 0.18	1.73 ± 0.07
Cone length, CL	1.46–3.14	3.46–4.24	3.22–4.01
	2.42 ± 0.03	3.09 ± 0.40	2.99 ± 0.40
Cone width, CW	3.05–4.71	3.05–4.71	3.20–4.95
	3.83 ± 0.03	3.83 ± 0.03	3.99 ± 0.03
BWOv/CW ratio	1.62–2.66	1.45–2.22	1.30–2.25
	2.11 ± 0.02	2.01 ± 0.02	2.00 ± 0.02
Oral sucker area, OSA	0.26–0.78	0.28–0.89	0.25–0.86
	0.49 ± 0.01	0.53 ± 0.01	0.55 ± 0.01
Maximum diameter of the oral sucker, OSmax	0.79–1.20	0.55–1.12	0.88–1.23
	1.01 ± 0.01	1.01 ± 0.10	1.05 ± 0.08
Minimum diameter of the oral sucker, OSmin	0.39–0.83	0.45–0.98	0.55–0.99
	0.61 ± 0.01	0.82 ± 0.09	0.82 ± 0.08
Ventral sucker area, VSA	1.39–2.21	1.39–2.21	1.40–2.10
	1.83 ± 0.02	1.83 ± 0.02	1.88 ± 0.02
Maximum diameter of the ventral sucker, VSmax	1.36–1.92	1.12–1.82	1.18–1.75
	1.61 ± 0.01	1.57 ± 0.14	1.54 012
Minimum diameter of the ventral sucker, VSmin	1.01–1.59	0.95–1.64	0.97–1.60
	1.44 ± 0.01	1.41 ± 0.15	1.38 ± 0.12
OSA/VSA ratio	0.13–0.47	0.23–0.57	0.27–0.64
	0.27 ± 0.01	0.38 ± 0.07	0.41 ± 0.06
Distance between anterior body end and ventral sucker, A-VS	1.40–3.48	2.61–3.87	2.39–3.63
	2.37 ± 0.03	3.33 ± 0.31	3.02 ± 0.27
Distance between oral sucker and ventral sucker, OS-VS	0.75–2.67	0.85–2.98	0.84–2.99
	1.76 ± 0.03	1.78 ± 0.03	1.79 ± 0.03
Distance between ventral sucker and vitelline gland union, VS-Vit	14.24–26.61	21.57–38.18	16.27–29.09
	20.96 ± 0.24	30.45 ± 3.76	22.53 ± 2.92
Distance between vitelline gland union and posterior body end, Vit-P	5.17–12.37	9.82–21.37	6.69–18.30
	8.52 ± 0.12	14.83 ± 2.50	11.84 ± 2.04
Distance between ventral sucker and posterior body end, VS-P	22.88–38.06	37.25–51.23	25.6–45.8
	29.48 ± 0.30	45.28 ± 3.25	34.3 ± 3.7
BL/VS-P ratio	1.02–1.27	1.04–1.13	0.97–1.08
	1.07 ± 0.003	1.07 ± 0.02	1.05 ± 0.02
Pharynx area, PhA	0.14–0.41	0.25–0.40	0.19–0.39
	0.26 ± 0.01	0.32 ± 0.01	0.25 ± 0.01
Pharynx length, PhL	0.50–1.01	0.48–1.04	0.71–1.21
	0.77 ± 0.01	0.86 ± 0.11	0.99 ± 0.10
Pharynx width, PhW	0.30–0.66	0.27–0.61	0.32–0.71
	0.46 ± 0.01	0.44 ± 0.07	0.48 ± 0.09
Testes area, TA	41.51–107.06	42.98–125.06	42.50–120.02
	75.96 ± 1.29	85.09 ± 1.30	85.01 ± 1.20
Testes length, TL	8.23–18.85	15.45–27.60	9.78–22.57
	14.32 ± 0.21	22.10 ± 3.51	15.73 ± 2.57
Testes width, TW	5.40–8.72	5.02–7.32	5.01–7.00
	6.90 ± 0.07	6.01 ± 0.06	5.99 ± 0.06
Testes perimeter, TP	30.22–56.13	31.33–57.56	31.33–57.56
	44.29 ± 0.51	44.45 ± 0.52	44.45 ± 0.52
Testes roundness, TR	1.48–2.76	1.48–2.66	1.50–2.69
	2.07 ± 0.03	2.10 ± 0.03	2.18 ± 0.03

**Table 4:** Polymorphic sites in sequence comparison of the nuclear rDNA whole intergenic region and in the ITS-1 and ITS-2 between haplotypes of *Fasciola hepatica* from Ecuador and haplotypes of genetically “pure” *F. gigantica* from African countries.

*Fasciola* spp. and Combined Haplotypes	Polymorphic Sites Intergenic Region (ITS-1, 5.8S, ITS-2)												
Positions	24	114	208	286	306		821	834	860	866	874	917	924
	Polymorphic sites ITS-1						Polymorphic sites ITS-2						
Positions	24	114	208	286	306		234	248	273	279	288	330	337
*F. hepatica* 1A	C	A	C	T	C		T	A	C	C	C	T	G
*F. hepatica* 2A	C	A	C	T	C		T	A	C	C	T	T	G
*F. hepatica* 1/2A *	C	A	C	T	C		T	A	C	C	C/T	T	G
*F. gigantica* 1A	T	T	T	A	T		C	A	T	T	C	-	A
*F. gigantica* 2A	T	T	T	A	T		C	C	T	T	C	-	A
*F. gigantica* 1/2A **	T	T	T	A	T		C	C/A	T	T	C	-	A

* = heterozygotic in position 874/288 not differentiating between *F. hepatica* and *F. gigantica*. ** = heterozygotic in position 834/248 not differentiating between *F. hepatica* and *F. gigantica* (also designed as H3A in Chougar et al. [26]. GenBank accession numbers for whole intergenic region: *F. hepatica* H1A = MG569980; *F. hepatica* H2A = MG569981; *F. gigantica* H1A = AJ853848.

**Table 5:** Nucleotide sequence (1533 bp-long) and protein sequence (511 aa-long) of the complete mtDNA *cox*1 gene of *Fasciola* *hepatica* populations studied from Ecuador compared to other haplotypes of the same species. Positions = numbers (to be read in vertical) refer to variable positions obtained in the alignment made with MEGA 7.0; . = identical. the Courier.

*Fasciola hepatica* Haplotypes	*cox*1 Nucleotide Sequence	COX1 Amino Acid Sequence	Nucleotide Composition (AT%)
Positions	111 1111111111 222333555 6777999112 2333444445 7145027467 2246023381 4666488992 2935985673 7670670788 0567325561	11444 01159 30469	
*F. hepatica*cox1-1 * cox1-H2-H69 ^	GGCGGTGAGA TCTCTGCATA GTTCAGTAAC tttttcagag cactcatgcg acctgacttw	LSVSN fpilf	
Fh-cox1-16 Fh-cox1-23 Fh-cox1-53 Fh-cox1-54 Fh-cox1-55 Fh-cox1-56 Fh-cox1-57 Fh-cox1-70 (new) Fh-cox1-5 ^§^ Fh-cox1-42 ^§^ Fh-AF216697 ^†^ Fh-M93388 ^‡^	.T........ ......T... .......... T......G.. C..TC.TG.. .......... .......... .A....T.C. ....G.C... .......... .AC...T... ....G.C... T......G.. C..TC.TG.. .........T .......... .A....T... ....G.CTT. T......G.. ...TC.TG.. .......... T......... ......T... .......... .......... .A....T... ....G..... ........AG ......T..G .......... T......G.. C.....T... ..CT...... ..TTTCA... ...T.ATG.. AC...A....	..... ..... ..... ..... ..... ....F ..... ..... ..... ..... ...L. FPI..	62.75 62.56 62.56 62.56 62.62 62.62 62.62 62.75 62.60 62.60 62.60 63.00

* = *F. hepatica cox*1 model represented by haplotype H1 from Spain; ^ other nucleotides appearing in these positions in haplotypes H2 to H69 (in these positions only the nucleotides of H1 or H2-H69 appear; w = only in position 1521 more than two different nucleotides appear) [17]; ^§^ = from Uruguay [43]; ^†^ = Geelong strain from Australia [43]; ^‡^ = from Utah, USA [43].

**Table 6:** Nucleotide sequence (903 bp-long) and protein sequence (300 aa-long) of the complete mtDNA *nad*1 gene of *Fasciola hepatica* populations studied from Ecuador compared to other haplotypes of the same species. Positions = numbers (to be read in vertical) refer to variable positions obtained in the alignment made with MEGA 7.0; . = identical.

*Fasciola hepatica* Haplotypes	*nad*1 Nucleotide Sequence	NAD1 Amino Acid Sequence	Nucleotide Composition (AT%)
Positions	22335667 88 2345097176 07 0663394523 14	7	
*F. hepatica* nad1-1 * nad1-H2-H51 ^	CACTTTTTTT AT tgtccacccc gc	A v	
Fh-nad1-2 Fh-nad1-6 Fh-nad1-14 Fh-nad1-23 Fh-nad1-42 Fh-nad1-43 Fh-nad1-12 ^§^ Fh-AF216697 ^†^ Fh-M93388 ^‡^	..TC...C.. .. ..T...C... .. .GT....... .. ..T...C..C .. ..TC...C.. .C ..TC..CC.. .C ..T....... .. TGT.C.C.C. G. ..T..AC..C ..	. . . . . . . V .	65.23 65.34 65.34 65.23 65.12 65.01 65.50 65.01 65.23

* = *F. hepatica nad*1 model represented by haplotype H1 from Spain; ^ = other nucleotides appearing in these positions in haplotypes H2 to H51 (in these positions only the nucleotides of H1 or H2-H51 appear) [17]; ^§^ = from Uruguay [43]; ^†^ = Geelong strain from Australia [43]; ^‡^ = from Utah, USA [43].

**Table 7:** DNA marker haplotypes distributed according to geographical origin, including comparison of Ecuador with other countries. Geographical data from other countries concern only complete sequences of each rDNA spacer and mtDNA gene.

DNA Marker	Haplotypes	Ecuador			Other Countries
		San Juan	Zapotillo	Macará	
ITS-2	FhITS2-1	sheep/cattle	cattle	cattle	Spain, France, Poland, Mexico, Venezuela,
					Peru, Bolivia, Uruguay, Argentina
	FhITS2-2	cattle			Spain, Andorra, Mexico, Bolivia, Uruguay
	FhITS2-1/2	sheep			
ITS-1	FhITS2-A	sheep/cattle	cattle	cattle	Spain, France, Poland, Mexico, Venezuela,
					Peru, Bolivia, Uruguay, Argentina
*cox*1	Fh*cox*1-16	sheep	cattle	cattle	Venezuela (cattle), Peru (sheep/cattle),
					Bolivia (sheep/cattle), Uruguay (cattle)
	Fh*cox*1-23	sheep			Mexico (cattle), Peru (cattle),
					Argentina (cattle), Chile (cattle)
	Fh*cox*1-53	cattle			Chile (cattle)
	Fh*cox*1-54	cattle			
	Fh*cox*1-55	cattle			Mexico (cattle)
	Fh*cox*1-56	sheep			
	Fh*cox*1-57	sheep			
	Fh*cox*1-70 new	sheep			
*nad*1	Fh*nad*1-2	sheep			Spain (sheep/cattle), Poland (bison), Venezuela (cattle), Peru
					(cattle), Bolivia (cattle), Uruguay (cattle),
					Argentina (sheep/cattle), Chile (cattle)
	Fh*nad*1-6	cattle			Spain (cattle)
	Fh*nad*1-14	sheep	cattle	cattle	Mexico (cattle), Venezuela (cattle), Peru (sheep/cattle), Uruguay (cattle), Argentina (cattle)
	Fh*nad*1-23	sheep			Mexico (cattle), Peru (cattle), Bolivia (sheep),
					Argentina (cattle), Chile (cattle)
	Fh*nad*1-42	cattle			
	Fh*nad*1-43	cattle			

## Data Availability

Datasets generated for this study are available on request to the corresponding author.
